# Supplementary material for: The Construction and Exploration of a Comprehensive MicroRNA Centered Regulatory Network in Foxtail Millet (Setaria italica L.)
Source: Front Plant Sci. 2022 May 6;13:848474. doi: 10.3389/fpls.2022.848474 (PMC9121102; doi:10.3389/fpls.2022.848474)
Supplement: Supplementary file 6 [file Data_Sheet_6.zip › Sit-miRN3023b_Seita.8G085200.1_3554_TPlot.pdf]

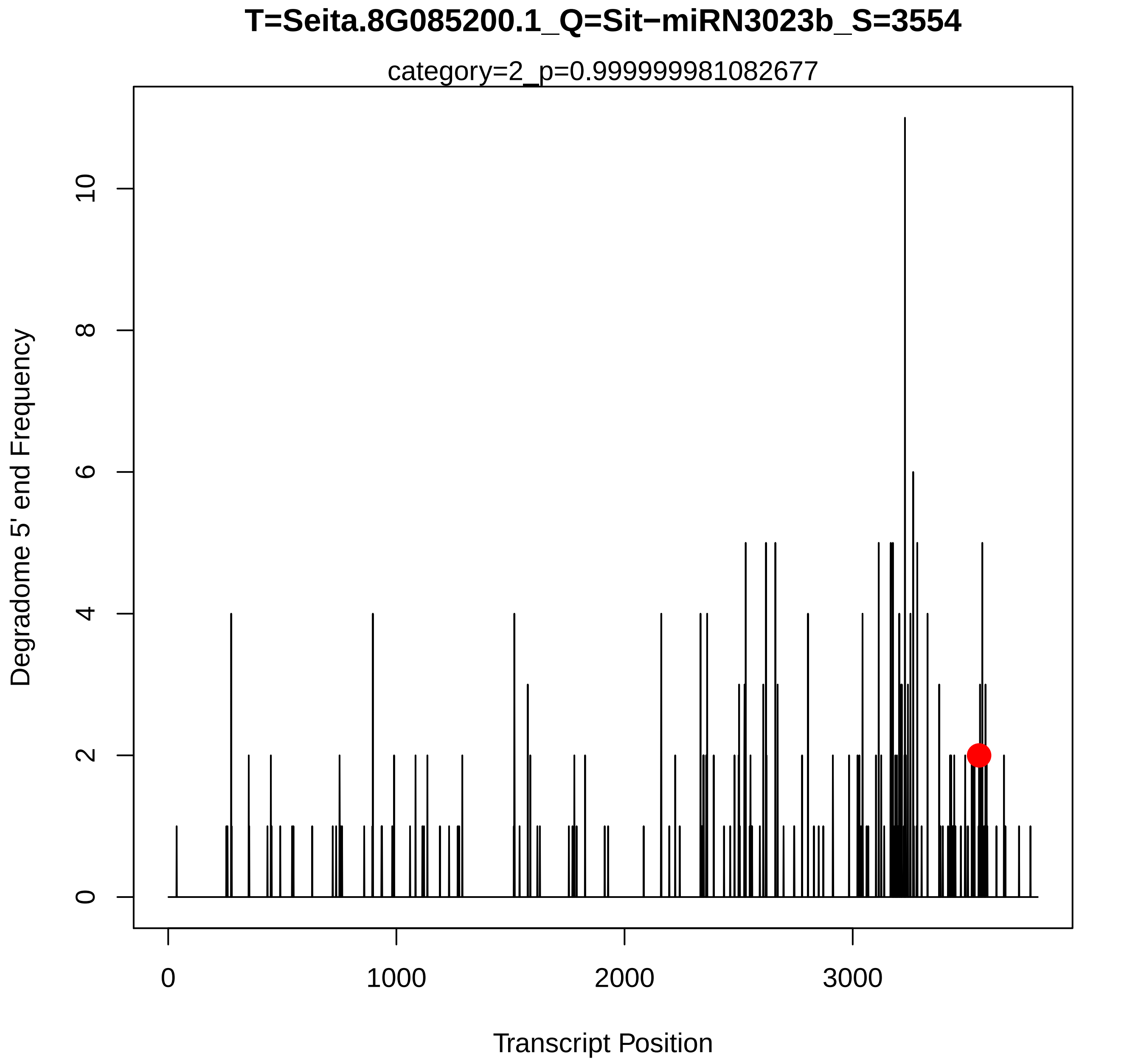
T=Seita.8G085200.1_Q=Sit−miRN3023b_S=3554
category=2_p=0.999999981082677
Degradome 5' end Frequency
0
2
4
6
8
10
0
1000
2000
3000
Transcript Position
